# Environmental Behavior, Toxicological Pathways, and Risk Assessment of Polycyclic Aromatic Hydrocarbons (PAHs): From Molecular Structure to Human Health

**DOI:** 10.3390/molecules31132211

**Published:** 2026-06-23

**Authors:** Joanna Harasym, Edyta Nizio

**Affiliations:** 1 Adaptive Food Systems Accelerator, Research Centre, Wroclaw University of Economics and Business, Komandorska 118/120, 53-345 Wroclaw, Poland; 2 Department of Biotechnology and Food Analysis, Wroclaw University of Economics and Business, Komandorska 118/120, 53-345 Wroclaw, Poland; 3 Department of Agroengineering and Quality Analysis, Wroclaw University of Economics and Business, Komandorska 118/120, 53-345 Wroclaw, Poland; edyta.nizio@ue.wroc.pl

**Keywords:** polycyclic aromatic hydrocarbons, environmental fate, chemical kinetics, toxicological mechanisms, bioaccumulation, bioremediation, risk assessment

## Abstract

Polycyclic aromatic hydrocarbons (PAHs) represent a major class of ubiquitous environmental pollutants, posing significant risks to ecosystems and human health due to their persistence, toxicity, and potential for bioaccumulation. This review provides a comprehensive synthesis of current scientific knowledge on PAHs, integrating insights from chemical kinetics, environmental fate, and toxicological mechanisms. The fundamental structural chemistry of PAHs and its direct influence on their physicochemical properties and environmental properties are discussed. The major anthropogenic and natural sources of PAHs are detailed, alongside the chemical kinetics behind their formation during incomplete combustion and their transformation in environmental media. Unlike previous reviews that address PAH sources, remediation, or health effects as separate topics, this review uniquely traces the mechanistic continuum from molecular formation kinetics through physicochemical partitioning and environmental transport to toxicological endpoints, providing a causally linked framework for understanding how structural properties ultimately determine biological outcomes. A central focus is placed on the environmental fate and transport of PAHs across atmospheric, aquatic, and terrestrial compartments, highlighting processes such as gas–particle partitioning, sediment accumulation, and long-range transport. The review further elucidates the complex toxicological pathways of PAHs, including metabolic activation to reactive intermediates, DNA adduct formation, oxidative stress, and their roles in carcinogenesis and other systemic health effects. The analysis reveals strong scientific consensus on the carcinogenic mechanism of parent PAHs via CYP450-mediated metabolic activation to diol-epoxide intermediates while identifying critical areas of uncertainty: the current regulatory framework based on 16 priority PAHs underestimates total carcinogenic risk by a factor of 2–5, mixture toxicology remains poorly characterized, and dose–response relationships for non-cancer endpoints (cardiovascular, neurodevelopmental, immunotoxic) lack the quantitative data needed for robust risk assessment. Finally, human exposure pathways and health risk characterization approaches are discussed, highlighting the need for cumulative, mixture-based assessment frameworks.

## 1. Introduction

Polycyclic aromatic hydrocarbons (PAHs) constitute a diverse class of ubiquitous organic contaminants characterized by two or more fused aromatic rings in linear, angular, or clustered configurations [1]. These inherently non-polar, planar molecules possess a hydrophobic structure that facilitates strong π-π stacking, leading to a high affinity for particulate matter and organic carbon in soils and sediments [1,2]. This molecular architecture, coupled with high molecular weight and exceptional thermostability, results in remarkable environmental persistence, recalcitrance to natural attenuation, and a strong tendency to bioaccumulate in organisms, posing long-term ecological and human health risks [3,4]. Their key physicochemical properties—low aqueous solubility, high sorptive affinity, and resistance to microbial degradation—severely limit bioavailability and promote accumulation in environmental sinks like soils, sediments, and biota. This not only prolongs persistence but also complicates remediation, as sorbed fractions become increasingly inaccessible to degradation processes. Furthermore, their lipid solubility enhances bioaccumulation potential through food chains, raising concerns about chronic exposure at higher trophic levels [5].

The global ubiquity of PAHs stems from continuous formation and release via both anthropogenic and natural processes, leading to their detection in all environmental media across diverse geographical regions [1]. This widespread distribution reflects multiple emission sources and the capacity for long-range atmospheric transport. Major regulatory agencies, including the U.S. EPA, have designated PAHs as priority pollutants due to their well-established toxicological profiles encompassing carcinogenicity, mutagenicity, and teratogenicity [1]. Sixteen PAHs are classified as U.S. EPA priority pollutants, with several, like benzo[a]pyrene, recognized as confirmed or probable human carcinogens by the International Agency for Research on Cancer (IARC). Their toxicological significance extends beyond cancer to include genotoxicity via DNA adduct formation, oxidative stress, immunotoxicity, and developmental neurotoxicity, reflecting complex interactions with biological systems [4]. The persistence of PAHs in environmental reservoirs like sediments facilitate long-term, chronic exposure, necessitating sustained monitoring and long-term remediation strategies.

The recognition of PAHs as hazards has evolved over the past century, with early observations dating back to the 18th century, linking soot and coal-tar exposure to cancer among chimney sweeps. Systematic environmental monitoring gained momentum in the 1970s–1980s with advances in analytical chemistry, particularly gas chromatography–mass spectrometry (GC-MS), enabling sensitive quantification and revealing global contamination. The U.S. EPA’s designation of 16 priority PAHs in the 1980s established a landmark regulatory framework widely adopted internationally. Recent decades have seen substantial progress in understanding PAH sources, fate, toxicology, and remediation, driven by advances in molecular biological tools and computational modeling. However, significant knowledge gaps remain, particularly regarding the toxicology of PAH mixtures, the environmental behavior of derivatives, and the influence of climate change on PAH dynamics [2,3].

Several recent reviews have addressed individual aspects of PAH science. Ukiwe et al. [1] focused primarily on degradation techniques, Kim et al. [3] concentrated on airborne PAHs and their health effects, and Patel et al. [4] provided a broad overview of sources, toxicity, and remediation approaches. Feng et al. [6] summarized environmental persistence and health risks without detailed kinetic analysis. However, none of these reviews systematically traced the causal chain linking molecular-level formation kinetics to physicochemical properties, environmental partitioning behavior, and ultimately toxicological outcomes. This fragmented treatment shows critical interdependencies—for example, how combustion kinetics determine congener profiles—which in turn impacts gas–particle partitioning, atmospheric lifetimes, deposition patterns, and the bioavailability that regulates metabolic activation and toxic potency. The present review addresses this gap by providing an integrated, mechanistic assessment that connects these domains within a single coherent picture, enabling readers to understand not only what happens to PAHs in the environment but why, based on quantitative structure–property–fate–toxicity relationships.

## 2. Structural Chemistry and Classification

**Figure 1:**
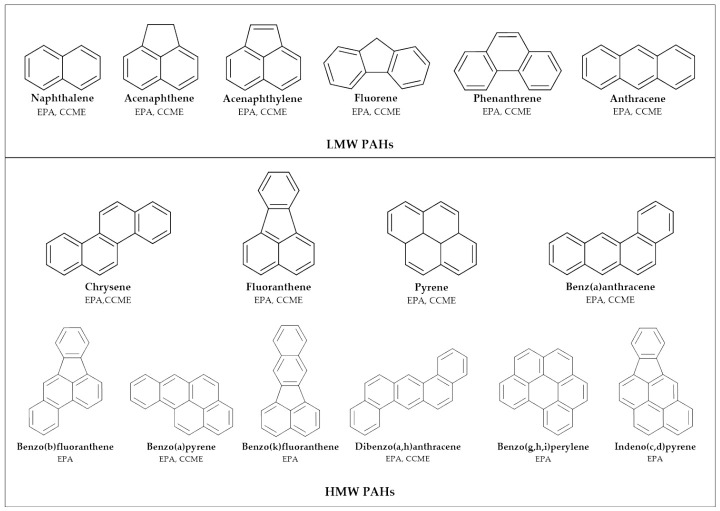
The 16 U.S. EPA priority parent PAHs—homocyclic compounds with fused benzene rings.

The physicochemical behavior, environmental fate, and toxicological profile of polycyclic aromatic hydrocarbons are inextricably linked to their molecular architecture, defined by the number and arrangement of fused aromatic rings. Understanding these structural characteristics is essential for predicting environmental behavior, assessing risks, and designing remediation strategies [1]. The 16 U.S. EPA priority parent PAHs—homocyclic compounds composed exclusively of fused benzene rings—are shown in Figure 1.

### 2.1. Molecular Architecture and Aromaticity

The defining feature of PAHs is two or more fused benzene rings in linear, angular, or clustered geometries [1]. This fusion creates extended π-electron systems that confer unique properties. PAHs are predominantly planar molecules due to sp^2^ hybridization, a geometry that facilitates strong π-π stacking interactions. This promotes the formation of ordered crystal structures, strong surface adsorption, and influences physical properties such as high melting points and low vapor pressures [1,2].

The extended conjugated system imparts exceptional stability, making PAHs resistant to chemical degradation and contributing to their environmental persistence. The electron-rich nature also makes them susceptible to electrophilic attack, forming the basis for both metabolic activation in biological systems and certain chemical remediation strategies. The planar geometry and strong π-π stacking capacity significantly contribute to their tendency to adsorb strongly onto particulate matter and organic carbon, especially in soil and sediment, sequestering them in environmental sinks and reducing bioavailability [1,2].

### 2.2. Classification Based on Molecular Weight and Ring Number

PAHs are systematically classified by the number of aromatic rings, a scheme that correlates with molecular weight and environmental behavior. The most widely adopted classification divides PAHs into low-molecular-weight (LMW) and high-molecular-weight (HMW) compounds, with the boundary typically at three to four rings [4].

#### 2.2.1. Low-Molecular-Weight PAHs

LMW PAHs contain two or three aromatic rings (e.g., naphthalene, phenanthrene) and molecular weights are generally below 200 g/mol [1,4]. They exhibit higher volatility and aqueous solubility compared to HMW PAHs, facilitating their presence in the gas phase and enabling long-range atmospheric transport. Their greater mobility results in more widespread dispersion but also more rapid natural attenuation through volatilization, photodegradation, and microbial degradation. Although being generally less carcinogenic than HMW PAHs, they can exhibit significant acute toxicity to aquatic organisms.

#### 2.2.2. High-Molecular-Weight PAHs

HMW PAHs contain four or more rings (e.g., pyrene, benzo[a]pyrene) and molecular weights typically exceed 200 g/mol [1,4]. They are characterized by greater molecular stability, drastically reduced aqueous solubility, very low vapor pressures, and markedly enhanced carcinogenic and mutagenic potentials. Their enhanced lipophilicity leads to strong binding to organic matter in sediments, soils, and biological tissues [2,3]. This strong sorption enhances persistence, promotes accumulation in environmental sinks, and facilitates bioaccumulation. The combination of persistence, bioaccumulation potential, and high toxicity establishes HMW PAHs as the compounds of greatest long-term concern.

### 2.3. Structure–Property Relationships

The physicochemical properties of PAHs exhibit systematic, predictable variations with molecular size. Key parameters—including aqueous solubility, vapor pressure, and partition coefficients—show highly linear relationships when plotted against molar mass or the number of aromatic rings [5]. Aqueous solubility decreases logarithmically, while the octanol–water partition coefficient (log K_ow), quantifying lipophilicity, increases systematically with ring number. These characteristics have a profound implication for environmental partitioning, bioavailability, and bioaccumulation potential [2,5]. Additionally, the vapor pressure decreases dramatically, critically influencing atmospheric transport. Such consistent behavior enables robust predictive modeling of PAH accumulation and transport [5]. Beyond size, specific geometric arrangements (e.g., bay regions and K-regions) influence chemical reactivity and are preferential sites for metabolic activation.

### 2.4. Priority PAHs and Regulatory Classifications

Given the hundreds of possible PAHs, regulatory attention focuses on a subset of particular concern. The most influential is the U.S. EPA’s list of 16 priority PAHs, which encompasses compounds from two to six rings (Table 1). Among these, seven are classified as probable human carcinogens, with benzo[a]pyrene often serving as an index compound for assessing carcinogenic potency via toxic equivalency factors (TEFs).

However, research suggests the 16 priority PAHs may not fully capture the risk from environmental mixtures, such as non-priority compounds, alkylated PAHs, and derivatives, can contribute substantially to overall toxicity. In response, some regulatory frameworks, like the European Union’s Water Framework Directive, have expanded their scope to include a broader spectrum of PAH-related compounds for comprehensive risk assessment [6,7,8].

## 3. Physicochemical Properties

The physicochemical properties of PAHs shape their environmental fate, transport, bioavailability, and toxicity (Table 2). These structure-dependent properties regulate how PAHs distribute among environmental compartments and are crucial for fate modeling, risk assessment, and remediation design.

Environmental fate prediction requires thermodynamically consistent property data [5]. Key parameters—vapor pressure, aqueous solubility (S_w_), octanol–water partition coefficient (log K_OW_), octanol–air coefficient (K_OA_), Henry’s law constant (K_AW_), and organic carbon sorption coefficient (K_OC_)—are interconnected (K_OW_ = K_OA_ × K_AW_), enabling robust fate modeling and property estimation [5].

Vapor pressure regulates gas–particle partitioning and volatilization, ranging c.a. 10 orders of magnitude and decreasing with molecular size: from c.a. 10 Pa for naphthalene (two rings) to c.a. 7 × 10^−7^ Pa for benzo[a]pyrene (five rings) at 25 °C. Temperature dependence follows the Clausius–Clapeyron relationship, producing seasonal concentration variation. LMW PAHs (2–3 rings) reside predominantly in the gas phase, enabling long-range transport but heightening degradation; HMW PAHs (4+ rings) are particle-bound, shielded from gas-phase reactions but susceptible to deposition. Aqueous solubility, which decreases systematically with molecular size, controls aquatic behavior, bioavailability, and groundwater transport: c.a. 31 mg/L for naphthalene, c.a. 0.0016 mg/L for benzo[a]pyrene, c.a. 0.06 μg/L for indeno[1,2,3-cd] pyrene; values rise modestly with temperature. The extremely low solubilities of HMW PAHs mean most “dissolved” PAHs are colloid- or DOM-associated, restricting aqueous transport and water-based remediation. PAHs are readily soluble in organic solvents, a property exploited analytically and in remediation [4].

Log K_OW_ is the most critical property for predicting partitioning, bioaccumulation, and toxicity [5], ranging from c.a. 3.4 (naphthalene) to >7.0 (coronene). It impacts affinity for soil/sediment organic carbon (log K_OC_ correlates with log K_OW_) [2] and is the principal bioaccumulation predictor, with maximum uptake in the “bioaccumulation window” of log K_OW_ c.a. 5–7—the lipophilicity range where compounds are hydrophobic enough to partition into biological membranes yet not so hydrophobic as to be sequestered by environmental sorbents [3,10]. Values > 7 reduce bioavailability through strong sorption and limited membrane permeability [3]. Log K_OW_ also correlates with baseline narcosis toxicity and is a key QSAR descriptor.

PAHs are solids at ambient temperature with melting points rising with molecular size (naphthalene 80 °C; benzo[a]pyrene 179 °C) owing to π–π stacking; sublimation is significant for LMW PAHs. Exceptional thermal stability (decomposition > 400–500 °C, reflecting aromatic stabilization energy) contributes to persistence and necessitates high-temperature thermal remediation. Sorption to soils and sediments is influenced by organic carbon content, particle size, and temperature [11]. Initial sorption is rapid, but slow diffusion into micropores and organic matter (“aging”, “sequestration”) creates fractions resistant to desorption and biodegradation. Temperature reduces sorption but can enhance hysteresis—desorption isotherms lying above adsorption isotherms—because conformational changes in organic matter or micropore entrapment make release more energy-demanding than uptake. Black carbon (and biochar) drives “super-sorption” through π–π interactions, drastically reducing bioavailability and mobility while prolonging persistence.

PAH–soot associations critically control environmental fate, long-range transport, and inhalation exposure [12,13,14]. Pyrogenic PAHs co-form with carbonaceous soot during combustion, generating strong sorptive bonds [14,15] and extremely slow desorption. Jonker et al. [12] used supercritical CO_2_ extraction (200 atm, 50 °C) on Amsterdam harbor soot, coal, activated carbon, and kerogen and reported desorption rate constants of 10^−7^ to 10^−5^ h^−1^ for the most tightly bound fractions, implying release timescales up to millennia. These extremes, however, derive from the strongest-binding end of the sorbent spectrum under laboratory conditions; environmental soot matrices are heterogeneous and undergo weathering, photochemical aging, and microbial colonization that modify surface properties and pore accessibility. Millennia-scale retention is plausible for the most recalcitrant fractions in undisturbed sediments but should not be generalized. Real-world behavior is different, rapid release of surface-adsorbed PAHs (hours–days), intermediate fractions (months–years), and essentially irreversible sequestration of PAHs occluded within condensed carbonaceous matrices [12,13]. Pyrogenic PAHs show stronger black carbon associations than petrogenic PAHs: increasing soot carbon content linearly decreases pyrogenic bioavailability while negligibly affecting petrogenic compounds, reflecting their combustion-derived carrier matrix [14]. Fine soot particles (≤2.5 μm) carrying adsorbed PAHs penetrate deeply into lung tissue [3] and enable long-range atmospheric transport [12,13]. Desorption rate constants of 10^−7^ to 10^−5^ h^−1^ indicate soot-bound PAHs are essentially unavailable for degradation, uptake, or aqueous transport [12], so conventional risk models likely overestimate bioavailable fractions [12,14].

Quantitative Structure–Property Relationships (QSPRs): It was observed that key parameters scale log-linearly with molar mass or ring number [5]. Log Kow rises c.a. 0.5–0.7 per ring; log(solubility) and log(vapor pressure) decline by comparable increments. These regularities support property estimation and fugacity/multimedia fate modeling [5]—a robustness that contrasts with the greater uncertainty of toxicological prediction for complex mixtures, where biological variables intervene.

Several uncertainties nonetheless warrant emphasis. First, reported physicochemical values vary substantially between studies, even for well-characterized compounds: HMW-PAH vapor-pressure measurements differ by up to an order of magnitude depending on technique (gas saturation, effusion, GC retention time), purity, and whether the value refers to the solid or supercooled liquid state [5,6]. Aqueous solubility data show similar scatter, particularly below 1 μg/L, where colloidal contamination, equilibration time, and analytical sensitivity all matter [6,7]. Second, although the Clausius–Clapeyron and van’t Hoff relationships describe temperature dependence well near 25 °C, extrapolation to environmental extremes introduces error: seasonal fluctuations shift gas–particle partitioning and sorption coefficients by factors of 2–10, and the constant-enthalpy assumption need not hold for all PAHs [5,10]. Third, QSPR predictive power, robust for the 16 priority parent PAHs, deteriorates for alkylated homologs, heterocyclic derivatives (dibenzothiophenes, carbazoles), and PAHs with more than six rings: limited calibration data, non-planarity from substituents, and steric effects on solvation/partitioning yield errors exceeding 0.5–1.0 log units in estimated K_OW_ or K_OC_ [5]. These uncertainties propagate into fate models, risk assessments, and remediation design, motivating continued experimental measurement across environmentally relevant temperature ranges, particularly for non-priority PAHs of growing concern.

## 4. Sources and Formation Mechanisms

The global distribution and environmental burden of polycyclic aromatic hydrocarbons (PAHs) result from diverse emission sources and formation pathways that include both natural and anthropogenic origins. PAH formation occurs through two principal mechanisms: pyrogenic processes involving high-temperature incomplete combustion of organic matter, and petrogenic processes associated with the geological formation and subsequent mobilization of fossil fuels [1]. Understanding the kinetics and mechanistic details of these processes is essential for predicting congener profiles, emission inventories, and ultimately the environmental and toxicological significance of PAH contamination.

Pyrogenic PAHs, constituting the majority of environmentally detected PAHs, are products of incomplete combustion or pyrolysis of organic carbon at elevated temperatures (typically 500–1200 °C). The underlying chemistry involves thermal decomposition of organic matter into small hydrocarbon radicals and fragments, which recombine through several well-characterized mechanisms to form stable polycyclic aromatic structures [1]. The relative contributions of these mechanisms are driven by temperature, fuel composition, oxygen availability, and residence time, producing characteristic congener profiles that serve as diagnostic source signatures.

### 4.1. Formation Kinetics and the HACA Mechanism

The hydrogen-abstraction–acetylene-addition (HACA) mechanism, first proposed by Frenklach and Wang [16], remains the most extensively studied pathway for PAH molecular growth in combustion environments. The HACA sequence proceeds through repetitive cycles consisting of two elementary steps: (1) hydrogen abstraction from an aromatic C–H bond, generating an aryl radical, followed by (2) acetylene (C_2_H_2_) addition to the radical site, ring closure, and hydrogen elimination to restore aromaticity and extend the π-conjugated system [16,17].

Rate-Limiting Step and Kinetic Parameters: Ab initio G3-type calculations by Kislov et al. [15] have generated comprehensive potential energy surfaces and rate constants for all elementary reactions in the HACA and Diels–Alder pathways. The hydrogen abstraction step is rate-limiting at combustion temperatures, with activation barriers of approximately 16–17 kcal/mol for abstraction by H atoms, compared to substantially lower barriers of c.a. 4 kcal/mol for abstraction by OH radicals [17]. The OH radical is thus predicted to be the fastest hydrogen abstractor from PAH molecules; even at 2500 K, the rate constant for H abstraction by atomic hydrogen remains approximately 34% lower than that for abstraction by OH [17]. These rate modified Arrhenius expressions of the form k = ATnexp(−Ea/RT), with representative values of A = 6.46 × 10^7^ cm^3^/mol∙s and Ea = 15.98 kcal/mol for the forward reaction [15,17]. The acetylene addition steps that follow are characterized by relatively low barriers and high exothermicity, confirming that ring growth is kinetically favorable once the radical site is generated [15].

Temperature Dependence and Pathway Competition: The HACA mechanism dominates PAH growth at high temperatures (>1500 K), where the rate of hydrogen abstraction is sufficiently rapid to sustain sequential ring additions [17]. Mebel et al. [17] provided temperature- and pressure-dependent rate coefficients for the HACA pathways from benzene to naphthalene, demonstrating that at temperatures above 1500 K and pressures near 1 atm, the HACA sequence efficiently produces naphthalene through both the Frenklach and Bittner–Howard routes. However, at intermediate temperatures (c.a. 1000–1300 K), radical recombination pathways become increasingly significant. Starting from naphthalene, the HACA-type synthesis of higher PAHs with exclusively six-membered rings (anthracene, phenanthrene) accounts for only 3–6% of the total product yield at combustion-relevant temperatures (1000–2000 K), whereas cyclopenta-fused PAHs constitute approximately 75% of the products [15]. This finding has important implications for predicting environmental congener profiles, as the HACA mechanism preferentially generates cyclopenta-fused species rather than the traditionally assumed benzenoid PAHs.

### 4.2. Alternative Formation Pathways: Radical Recombination, Diels–Alder, and Resonance-Stabilized Radicals

Diels–Alder [4+2] cycloaddition has long been proposed as an alternative PAH growth mechanism. However, detailed first-principle calculations demonstrate that Diels–Alder pathways cannot compete with HACA even at the highest combustion temperatures, due to substantially higher activation barriers and consequently lower rate constants [15]. For example, barrier heights for Diels–Alder cycloadditions exceed those of the corresponding HACA steps by 15–25 kcal/mol, rendering these pathways kinetically negligible under typical combustion conditions [15]. Nevertheless, Diels–Alder reactions may contribute to specialized environments with high diene concentrations or under catalytic conditions.

Several alternative mechanisms have been identified that compete with or complement the HACA pathway. The hydrogen-abstraction–vinyl-radical-addition (HAVA) pathway has been shown to be more efficient than HACA for producing PAHs during aliphatic hydrocarbon pyrolysis at moderate temperatures (c.a. 1300 K) [18]. The phenyl addition/cyclization (PAC) mechanism is considered the most efficient pathway for PAH growth, while the methyl addition/cyclization (MAC) mechanism occupies an intermediate position between PAC and HACA in terms of efficiency [18]. The carbon-addition–hydrogen-migration (CAHM) mechanism has also been proposed as competitive with HACA at lower temperatures, though detailed kinetic analysis by Mebel et al. suggests that HACA remains substantially faster than CAHM under post-flame conditions [17]. The relative importance of these competing pathways is sensitive to temperature, pressure, fuel type, and local radical concentrations, underscoring the complexity of predicting PAH congener distributions from first principles.

A significant recent advance is the discovery by Johansson et al. [19] that resonance-stabilized radicals (RSRs) play a central role in soot inception and rapid PAH growth. Unlike conventional radicals, RSRs possess unpaired electrons that participate in π-conjugation across the molecular framework, conferring stability while maintaining sufficient reactivity. These radicals react with other hydrocarbon species to form covalently bound complexes that promote further growth by regenerating resonance-stabilized radicals through low-barrier hydrogen-abstraction and hydrogen-ejection reactions [19].

The clustering of hydrocarbons by radical chain reaction (CHRCR) mechanism provides a pathway for covalently bound PAH clusters to form at temperatures where physical condensation of individual PAHs would be thermodynamically unfavorable. This mechanism resolves a longstanding puzzle: conventional HACA kinetics alone are insufficiently rapid to explain the experimentally observed rates of soot inception [19]. The lower-limit estimate for reaction collision efficiency (α) for these low-barrier reactions involving PAHs and radicals is approximately 0.01, indicating that a substantial fraction of radical–molecule collisions lead to productive covalent bond formation.

### 4.3. Atmospheric Transformation: Oxygenated and Nitrated PAH Formation

Following emission, PAHs undergo atmospheric transformation reactions with OH radicals, NO_3_ radicals, O_3_, and NO_2_, producing oxygenated (oxy-PAH) and nitrated (nitro-PAH) derivatives that are often more mutagenic and carcinogenic than the parent compounds [20,21].

In the gas phase, PAHs react predominantly with hydroxyl radicals, with rate coefficients on the order of 10–11 cm^3^/molecules at 298 K [20]. Representative measured rate constants (in units of 10–12 cm^3^/s) include: naphthalene, 23; acenaphthene, 58; fluorene, 13; phenanthrene, 27; anthracene, 190; and fluoranthene, 11 [21]. Rate constants translate to atmospheric lifetimes ranging from a few hours (anthracene) to approximately one day (fluoranthene) under typical OH concentrations of c.a. 10^6^ molecules cm^−3^. The pyrene lifetime under atmospheric OH conditions is estimated to be approximately 5.5 days [21]. These data demonstrate that LMW gas-phase PAHs are efficiently removed by OH-initiated oxidation, while HMW PAHs, being predominantly particle-bound, are shielded from gas-phase reactions.

Nitrated PAHs form through two distinct atmospheric pathways: (1) daytime OH radical-initiated addition to the PAH ring, followed by reaction with NO_2_ and loss of water; and (2) nighttime NO_3_ radical addition followed by reaction with NO_2_ and loss of HNO_3_ [20,21]. The isomer profiles produced by these two pathways are diagnostically distinct: the daytime OH pathway generates 2-nitrofluoranthene and 2-nitropyrene as the dominant products, while the nighttime NO_3_ pathway produces different positional isomers (e.g., 2- and 4-nitropyrene) [21]. Since neither 2-nitrofluoranthene nor 2-nitropyrene are found in direct combustion emissions, their atmospheric ubiquity provides strong evidence for in situ photochemical formation as the dominant source of ambient nitro-PAHs [20]. The International Agency for Research on Cancer classifies 1-nitropyrene as probably carcinogenic to humans, and several nitro-PAH derivatives exhibit mutagenic potencies exceeding those of the parent compounds.

Particle-associated PAHs react with NO_2_ and OH through heterogeneous processes at rates substantially lower than gas-phase reactions. Experimental studies using diesel particulate exhaust (NIST SRM 1650a) demonstrate that adsorbed PAHs are approximately four orders of magnitude more reactive with OH than with NO_2_, confirming OH as the dominant atmospheric oxidant for particulate PAH degradation [22]. This finding has important implications: the viscous organic matrix of combustion aerosols creates diffusion limitations that shield interior PAH molecules from oxidative attack, substantially prolonging atmospheric lifetimes of particle-bound HMW PAHs compared with predictions from gas-phase kinetics alone [23].

### 4.4. Influence of Oxygen Availability on Product Distributions

Oxygen availability exerts a critical influence on the balance between parent PAH formation and the generation of oxygenated derivatives during combustion and atmospheric processing. Under fuel-rich, oxygen-limited combustion conditions, the pyrolytic fragmentation of organic matter favors the radical-mediated assembly of parent PAHs through the HACA and related mechanisms described above. As oxygen availability increases, competing oxidation pathways become significant, diverting reactive intermediates toward oxygenated products including quinones, aldehydes, and carboxylic acids rather than continued ring growth [24].

In atmospheric environments, the interplay between oxidant concentrations (OH, O_3_, NO_3_) and PAH reactivity determines the steady-state partitioning between parent compounds and their transformation products. The ozonolysis of particle-associated PAHs is particularly complex; Zhou et al. [23] demonstrated that reactive uptake by ozone leads to the formation of viscous surface crusts composed of oxidation products, which act as protective diffusion barriers. This creates reaction kinetics markedly escaping from simple pseudo-first-order models, as the bulk PAH pool becomes increasingly shielded over time. The practical consequence is that models not accounting for this physical shielding effect systematically underestimate the atmospheric persistence and long-range transport potential of HMW PAHs [23].

### 4.5. Well-Established Aspects Versus Debated Issues

The mechanistic understanding of PAH formation chemistry encompasses both well-established aspects supported by converging experimental and computational evidence, and areas of active debate where significant uncertainties remain.

These well-established aspects include the following:

1. The general HACA sequence of hydrogen abstraction followed by acetylene addition and ring closure as a viable route for sequential PAH growth at high temperatures is supported by extensive ab initio calculations, flame measurements, and kinetic modeling [15,16,17].
2. Hydrogen abstraction is the rate-limiting step, with activation barriers of c.a. 16–17 kcal/mol for H-atom abstraction and c.a. 4 kcal/mol for OH–radical abstraction [15,17].
3. The Diels–Alder mechanism is kinetically inferior to HACA at combustion temperatures due to substantially higher barriers [15].
4. Gas-phase PAH oxidation by OH proceeds with rate constants on the order of 10–11 cm^3^/mol∙s, with OH being the dominant atmospheric oxidant for both gas-phase and particle-associated PAHs [15].

Debated issues include:

1. The relative contribution of HACA versus alternative mechanisms (HAVA, PAC, MAC, CHRCR) under different combustion regimes remains quantitatively uncertain, particularly at intermediate temperatures (1000–1500 K) where multiple pathways contribute comparably [17,18,19].
2. The role of resonance-stabilized radicals in soot inception, supported by experimental evidence [19], awaits comprehensive incorporation into predictive kinetic models across a wider range of flame conditions.
3. The branching between six-membered ring (benzenoid) and five-membered ring (cyclopenta-fused) PAH products during HACA growth is not fully resolved; computational predictions of c.a. 75% five-membered ring products [15] are not consistently reflected in environmental congener profiles, suggesting that additional pathways contribute to benzenoid PAH formation.
4. The extent to which heterogeneous diffusion limitations and surface crust formation alter atmospheric PAH lifetimes remains difficult to parameterize in global transport models, creating significant uncertainty in long-range transport predictions for HMW PAHs [23].

### 4.6. Anthropogenic and Natural Sources

#### 4.6.1. Anthropogenic Pyrogenic Sources

Anthropogenic activities are the predominant sources of pyrogenic PAHs. Key sources include industrial processes such as aluminum production, coke production, coal gasification, and iron and steel manufacturing, which operate at temperatures conducive to PAH formation and often handle PAH-rich materials like coal tar [1,25]. Vehicular emissions from both gasoline and diesel engines represent ubiquitous urban sources, emitting PAHs in both gas and particulate phases [25,26]. Residential combustion of solid fuels (wood, coal) for heating constitutes a major seasonal source, especially under suboptimal combustion conditions where oxygen-starved flames favor pyrolytic PAH formation [1,25]. Additional contributions arise from waste incineration, tobacco smoking, and high-temperature food preparation (grilling, smoking), which provide both environmental PAH burdens and direct human exposure pathways [1,25,26,27].

#### 4.6.2. Natural Pyrogenic and Petrogenic Sources

Wildfire is the primary natural pyrogenic source. Biomass burning, particularly during the smoldering phase, generates substantial quantities of PAHs. Post-fire runoff mobilizes PAHs from ash and carries charred organic matter into aquatic systems, creating persistent contamination pulses that can rival chronic anthropogenic inputs in fire-prone watersheds [2]. Volcanic activity contributes to minor, localized emissions.

Petrogenic PAHs are naturally occurring compounds formed over geological timescales through diagenesis of organic matter and are intrinsically present in fossil fuels including crude oil and coal [1]. Unlike pyrogenic PAHs, which are enriched in parent (unsubstituted) compounds, petrogenic profiles are typically dominated by alkylated homologs. Environmental inputs occur through oil spills and chronic petroleum releases [28,29,30], coal mining and processing [31,32,33], and volatilization and leaching from asphalt and road materials [34,35,36].

#### 4.6.3. Source Apportionment

Distinguishing among PAH sources is essential for environmental management. Source apportionment relies on characteristic differences in congener profiles. Diagnostic ratios of specific isomers (e.g., phenanthrene/anthracene, fluoranthene/pyrene) help discriminate between pyrogenic and petrogenic origins, though weathering can alter ratios post-emission. Multivariate statistical methods including principal component analysis (PCA) and positive matrix factorization (PMF) use the full congener profile for quantitative source apportionment. Stable carbon isotope ratios (δ^13^C) of individual PAHs provide a powerful complementary tool [25], as different fuels and formation processes impart distinct isotopic signatures that are more resistant to post-emission alteration.

## 5. Post-Emission Transformation and Degradation Kinetics

The persistence of PAHs in the environment is ultimately controlled by the rates at which they are transformed and degraded once released. These post-emission processes—photochemical, aqueous-phase oxidative, and biological—operate over much longer timescales than combustion chemistry and across all environmental compartments.

### 5.1. Photochemical Transformation

Photochemical transformation is a major abiotic degradation pathway in sunlit environments. PAHs absorb UV and visible light, undergoing direct photolysis or reaction with oxygen to form oxidized products such as quinones and epoxides. Photolysis kinetics depend on light intensity, the PAH-specific absorption spectrum, and the environmental matrix. Indirect photolysis mediated by reactive species such as hydroxyl and singlet oxygen, generated from photosensitizers—notably dissolved organic matter (DOM)—frequently proceeds more rapidly than direct photolysis [23,37]. In particulate matter, the multiphase reactivity of PAHs with O_3_ is heavily influenced by phase behavior and diffusion limitations: viscous oxidation product crusts form on particles and shield the underlying PAH pool from further oxidation. The resulting pseudo-first-order kinetics differs markedly from gas-phase predictions and substantially prolongs atmospheric lifetimes of HMW PAHs [38].

### 5.2. Aqueous and Chemical Oxidation

In aqueous systems, chemical oxidation using permanganate, persulfate, or Fenton-type reagents is exploited in engineered remediation. Advanced oxidation processes (AOPs) generate hydroxyl radicals capable of mineralizing PAHs, although complete mineralization is typically slow and partial-oxidation products may themselves be of toxicological concern. Hydrolysis is generally insignificant for parent PAHs, owing to their stability and absence of hydrolyzable functional groups [6,38,39].

### 5.3. Microbial Degradation

Microbial degradation is the dominant long-term degradation pathway in soils, sediments and surface waters. Bacteria primarily use ring-hydroxylating dioxygenases to incorporate two oxygen atoms into the aromatic ring, generating cis-dihydrodiols [40]. These intermediates are further oxidized to catechols, which undergo ring cleavage before entering central metabolic pathways for mineralization [41]. Specialized marine bacteria such as *Cycloclasticus* possess diverse dioxygenase systems that enable degradation of a wide range of PAHs and play a pivotal role in marine oil-spill bioremediation [41,42]. Fungal degradation employs non-specific extracellular ligninolytic enzymes (laccases, peroxidases) that generate aromatic radical cations, leading to oxidation, polymerization or coupling to soil organic matter; fungal PAH transformation is frequently a co-metabolic process [43]. Across all biological systems, bioavailability—controlled by sorption to organic matter and black carbon, by aging, and by micropore entrapment—is the dominant control on apparent degradation rate, often masking the intrinsic enzymatic kinetics.

## 6. Environmental Fate and Transport

**Figure 2:**
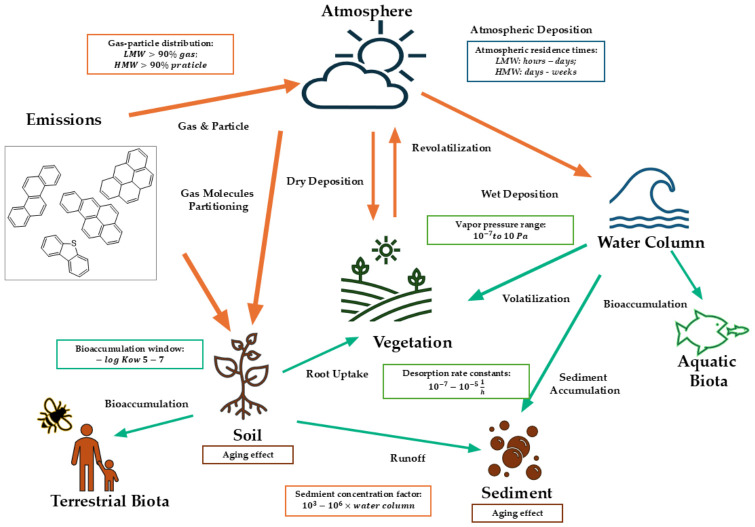
Environmental fate and transport pathways of PAHs. Orange—air pathway; green—water/soil pathway.

The environmental distribution and persistence of PAHs are ruled by complex physical, chemical, and biological processes across air, water, soil, and sediment compartments (Figure 2).

### 6.1. Atmospheric Processes and Partitioning

In the atmosphere, PAHs partition between gaseous and particulate phases, a process critically influenced by molecular weight, vapor pressure, and temperature [1]. Low-molecular-weight (LMW) PAHs (2–3 rings) reside predominantly in the gas phase, facilitating long-range transport but making them susceptible to rapid photochemical degradation [44]. High-molecular-weight (HMW) PAHs (4+ rings) are primarily bound to aerosols, protecting them from gas-phase oxidation but subjecting them to deposition. Intermediate PAHs exhibit dynamic, temperature-dependent partitioning [45]. While models like Junge–Pankow describe this equilibrium, kinetic limitations and aerosol viscosity can cause deviations [46].

Volatility and atmospheric residence times enable PAHs to undergo long-range transport, contaminating remote regions like the Arctic and Antarctic [47]. ‘Global fractionation’ occurs, where more volatile LMW PAHs travel farther than HMW PAHs, leading to congener profile gradients [48]. Climate change may alter these patterns by affecting temperatures, circulation, and the ‘grasshopper effect’ of remobilization. PAHs are transferred to ecosystems via dry deposition (direct settling) and wet deposition (precipitation scavenging), with the latter causing episodic ‘first flush’ pulses of contamination [49].

### 6.2. Aquatic Systems

PAHs enter aquatic environments through multiple pathways, including atmospheric deposition, surface runoff, riverine transport, groundwater discharge, and direct industrial/municipal effluents [2]. Wildfires are a significant and growing source, as post-fire runoff mobilizes PAHs from ash and carries charred biomass into water bodies, creating persistent sediment contamination [2]. In the water column, the strong lipophilicity of PAHs drives their rapid association with dissolved organic matter (DOM) and suspended particulate organic carbon (POC), controlling their transport through settling and resuspension [50,51,52].

Sediments are a major long-term sink for PAHs, with concentrations often 10^3^–10^6^ times higher than in the water column [3]. This accumulation concentrates toxicity in benthic ecosystems [2]. Over time, ‘aging’ processes—such as diffusion into micropores and association with black carbon—reduce the bioavailability of sediment-bound PAHs, limiting both degradation and uptake but contributing to persistence. Disturbances (e.g., dredging, floods) can remobilize these sequestered PAHs [53,54,55].

Aquatic organisms accumulate PAHs via gills, ingestion, and dermal contact. Bioaccumulation potential correlates with Kow, but efficient metabolic systems in vertebrates limit net accumulation. Biomagnification through food webs is generally limited due to this metabolic breakdown, although trophic transfer does occur, creating exposure risks for both wildlife and humans [56,57,58].

### 6.3. Terrestrial Ecosystems

PAHs enter soils through atmospheric deposition, runoff, spills, and the application of waste. The presence of black carbon or biochar enhances sorption, immobilizing PAHs and creating long-term contamination resistant to natural attenuation [59,60,61].

Plants interact with PAHs through three main pathways:

1. Root uptake—Generally limited for HMW PAHs due to strong soil sorption but can be significant for LMW compounds. Plants can stimulate microbial degradation in the rhizosphere (phytostimulation) [62].
2. Foliar uptake—A major pathway, where gas-phase PAHs diffuse through cuticles and particle-bound PAHs deposit on leaves. This is particularly important in urban areas, as it introduces PAHs into terrestrial food webs [63].
3. Vegetation–atmosphere exchange—A bidirectional process where plants can also revolatilize PAHs, acting as a secondary atmospheric source. This exchange varies with temperature, plant physiology, and atmospheric concentrations [64].

## 7. Toxicological Mechanisms

**Figure 3:**
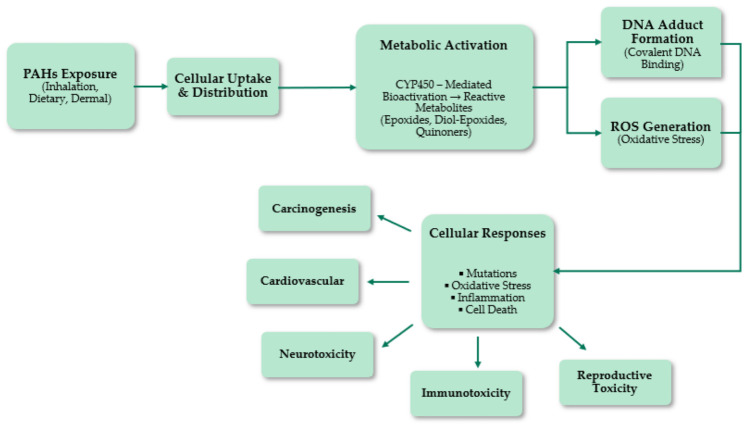
Schematic representation of PAH toxicological mechanisms showing the progression from exposure to uptake to metabolic activation (CYP450-mediated bioactivation to reactive metabolites) to molecular effects (DNA adduct formation, ROS generation) to cellular responses (mutations, oxidative stress, inflammation) to adverse outcomes (carcinogenesis, cardiovascular effects, neurotoxicity, immunotoxicity, reproductive toxicity).

The toxicity of polycyclic aromatic hydrocarbons (PAHs) results not from the parent compounds themselves, which are relatively inert, but from their metabolic activation into reactive intermediates that damage cellular macromolecules, leading to mutagenesis, carcinogenesis, and other adverse effects. Figure 3 presents a schematic representation of the major toxicological mechanisms through which PAHs exert their adverse effects, illustrating the progression from exposure and metabolic activation through molecular damage, cellular responses, and ultimately organ-specific toxicity and disease outcomes. This integrated view illustrates the various pathways through which PAHs impact human health.

### 7.1. Metabolic Activation and Bioactivation Pathways

The critical first step in PAH toxicity is enzymatic oxidation by Phase I enzymes, particularly cytochrome P450 (CYP) isoforms CYP1A1, CYP1A2, and CYP1B1. These enzymes are often induced by PAH exposure via the aryl hydrocarbon receptor (AhR) pathway [1]. The bioactivation process involves sequential oxidations: initial CYP-mediated reactions form arene oxides (epoxides), which can rearrange to phenols or be hydrolyzed by epoxide hydrolase to form trans-dihydrodiols. These dihydrodiols can be further oxidized to highly electrophilic diol-epoxides, the ultimate carcinogenic metabolites for many PAHs [1]. For benzo[a]pyrene (BaP), the prototypical carcinogenic PAH, this pathway generates (+)-benzo[a]pyrene-7,8-diol-9,10-epoxide (BPDE), which preferentially forms adducts with DNA. The balance between this bioactivation and Phase II detoxification enzymes (e.g., glutathione-S-transferases, GSTs), which conjugate metabolites for excretion, determines the net toxic outcome and varies between individuals and species [65,66,67].

### 7.2. Genotoxicity and DNA Adduct Formation

PAHs exert their genotoxicity primarily through the formation of DNA adducts. The electrophilic diol-epoxides covalently bind to nucleophilic sites on DNA bases, particularly guanine and adenine, forming stable or depurinating adducts [18]. Stable adducts, if unrepaired by nucleotide excision repair (NER), can lead to replication errors, resulting in base substitutions (e.g., G to T transversions) or frameshift mutations. Depurinating adducts create abasic sites that are also mutagenic. The mutation pattern is not random; hotspots often coincide with critical sequences in tumor suppressor genes, such as TP53, directly linking PAH exposure to cancer initiation. PAH-DNA adducts serve as crucial biomarkers of biologically effective dose, bridging external exposure with internal biological effect within the “exposome” concept [18,68,69,70].

### 7.3. Oxidative Stress and Reactive Oxygen Species

Beyond direct DNA damage, PAHs induce oxidative stress through multiple mechanisms, including the redox cycling of quinone metabolites, uncoupling of electron transport during CYP metabolism, mitochondrial dysfunction, and activation of NADPH oxidases. This generates reactive oxygen species (ROS), such as superoxide and hydroxyl radicals, leading to lipid peroxidation, protein oxidation, and oxidative DNA damage. This oxidative stress depletes antioxidants, such as glutathione, triggering cell death pathways (apoptosis and necrosis) and promoting chronic inflammation and carcinogenesis. Present research indicates that BaP, particularly when co-exposed to UV radiation, can disrupt immunometabolic pathways, such as glutaminolysis, which supplies glutamate for the synthesis of glutathione. This depletion exacerbates oxidative damage and can promote ferroptosis, an iron-dependent form of cell death [23,71,72,73].

### 7.4. Carcinogenicity and Tumor Formation

The carcinogenicity of PAHs is well-established through epidemiological and mechanistic studies. Cancer initiation begins with DNA adduct-induced mutations in proto-oncogenes and tumor suppressor genes. However, cancer is a multistep process requiring promotion and progression–clonal expansion of initiated cells, additional mutations, evasion of apoptosis, and angiogenesis. PAH exposure contributes to these later stages through sustained DNA damage, chronic inflammation, and immunosuppression. Occupational studies (e.g., coke oven, aluminum workers) provide strong evidence for PAHs causing lung, skin, and bladder cancers. Quantifying the risk from environmental (low-dose, chronic) exposure is more challenging but contributes significantly to the population cancer burden [74,75,76].

### 7.5. Organ-Specific and Systemic Toxic Effects

PAH toxicity extends beyond cancer to affect multiple organ systems:

1. Cardiovascular toxicity—Linked to atherosclerosis, myocardial infarction, and arrhythmias. PAHs like phenanthrene can disrupt cardiac ion channels, slowing conduction and increasing arrhythmia susceptibility [77].
2. Neurodevelopmental toxicity—Prenatal exposure is linked to reduced cognitive function, attention deficits, and behavioral problems in children, likely via oxidative stress, neurotransmitter disruption, and endocrine interference [78].
3. Reproductive and developmental toxicity—Effects include reduced sperm quality, altered estrous cycles, spontaneous abortion, low birth weight, and congenital abnormalities, potentially mediated by endocrine disruption [79].
4. immunotoxicity—can manifest as immunosuppression (reducing infection resistance) or inappropriate immune activation (promoting inflammation, allergy, autoimmunity). The immune system is highly sensitive to PAHs [80].
5. respiratory effects—inhalation exposure is associated with reduced lung function, asthma exacerbation, and COPD, due to direct irritation, inflammation, and oxidative stress in lung tissue [81].

## 8. Human Exposure and Health Risk Assessment

**Figure 4:**
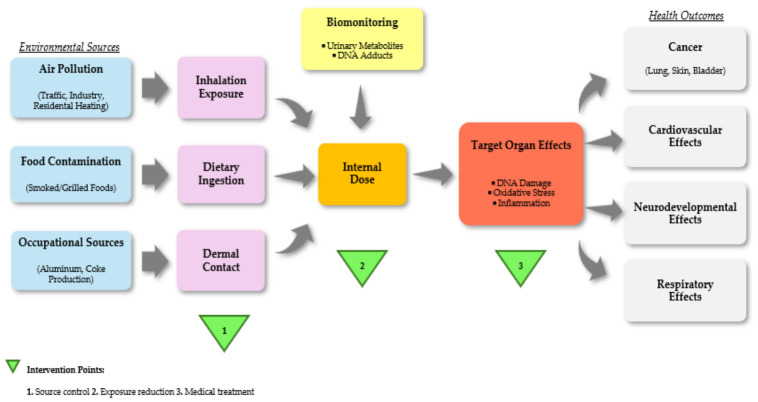
Human PAH exposure and health risk assessment. The diagram shows environmental sources (air pollution, food contamination, occupational sources); exposure routes (inhalation, dietary ingestion, dermal contact); biomonitoring (urinary metabolites, DNA adducts); internal dose and target organ effects to health outcomes (cancer, cardiovascular, neurodevelopmental, respiratory effects). Intervention points for risk reduction are indicated at multiple stages.

Understanding human exposure pathways and accurately assessing health risks are essential for protecting public health and guiding regulatory action. Exposure varies dramatically based on occupation, lifestyle, geography, and socioeconomic status (Figure 4).

### 8.1. PAHs Exposure Routes

Inhalation is a primary exposure route, especially in urban/industrial settings with emissions from vehicles, industry, and residential heating [1]. Indoor air pollution from solid fuel cooking and heating, as well as tobacco smoke, is also significant. Occupational exposures (e.g., coke production, firefighting) can far exceed ambient levels. Exposure magnitude depends on air concentration, breathing rate, and time–activity patterns. Urban residents, commuters, and children (due to higher breathing rates and time spent near the ground) often experience disproportionate exposure [82,83,84].

Diet is a critical exposure source for the general population. PAHs contaminate food through environmental deposition, processing, and high-temperature cooking. Grilled and smoked meats/fish have particularly high levels. Studies globally, from Southern Nigeria to Europe, show dietary exposure can rival or exceed inhalation for non-smokers, especially where traditionally prepared foods are consumed heavily [3]. Cooking methods drastically influence PAH content; boiling and steaming produce minimal PAHs compared to grilling or charring [85,86,87].

Dermal contact with contaminated soil, dust, coal-tar products, or occupational materials constitutes another pathway. While intact skin is a good barrier, prolonged contact, especially with LMW PAHs or damaged skin, allows significant absorption. Occupational dermal contact historically caused elevated skin cancer rates. Environmental exposure, particularly for children, can contribute to total body burden. Co-exposure to UV radiation enhances both skin permeability and PAH toxicity [88,89,90].

### 8.2. Biomonitoring and Exposure Assessment

Biomonitoring measures chemicals or their metabolites in biological tissues, providing integrated exposure assessment across all routes. Urinary hydroxylated metabolites (OH-PAHs) are the most practical biomarkers. 1-Hydroxypyrene (1-OHP) is widely used, but panels of multiple metabolites (e.g., hydroxyfluorenes, hydroxyphenanthrenes) provide a more comprehensive picture and better source apportionment, such as distinguishing tobacco smoke exposure [91,92]. These metabolites reflect recent exposure (24–48 h) and require correction for urine dilution. PAH-DNA adducts in blood lymphocytes represent a biologically effective dose (71), reflecting not just exposure but also metabolic activation and DNA damage, potentially offering a more direct link to cancer risk. However, their analysis is complex. PAH–protein (e.g., albumin) adducts integrate exposure over a longer period (weeks to months) and can serve as a surrogate for genotoxic dose [93,94].

### 8.3. Health Risk Characterization

Cancer risk for PAH mixtures is typically assessed using the toxic equivalency factor (TEF) approach, where the potency of individual PAHs is expressed relative to benzo[a]pyrene (BaP). The sum of concentration × TEF products gives a BaP-equivalent (BaPeq) concentration, which is used with cancer slope factors to calculate incremental lifetime cancer risk. Regulatory agencies often deem risks <10^−6^ acceptable and >10^−4^ unacceptable [75,95].

A major limitation is that regulatory focus on the 16 priority PAHs may underestimate total risk. Gas-phase PAHs and non-priority compounds (alkylated PAHs, derivatives) can contribute substantially to carcinogenic potency, meaning risks calculated from only 16 PAHs may be 2–5 times too low [2,96,97]. Non-cancer effects (cardiovascular, developmental, respiratory) are increasingly recognized and may occur at lower exposure levels than those required for carcinogenesis. Quantitative risk assessment for these endpoints is challenging due to limited dose–response data and a lack of established reference doses. Developmental neurotoxicity is a critical concern, but translating epidemiological associations into quantitative risk metrics remains difficult [98].

### 8.4. Regulatory Standards and Guidelines

Regulatory standards for PAHs vary internationally and by medium: air—often regulated via annual average BaP concentrations (e.g., EU target value: 1 ng/m^3^); occupational—permissible exposure limits (PELs) are set for specific PAH-containing materials; soil and water—cleanup standards and quality criteria vary by jurisdiction and land use, with residential standards being most stringent; food—maximum levels are set for BaP and sometimes the ‘PAH4’ (BaP, benz[a]anthracene, benzo[b]fluoranthene, chrysene) in various food categories, balancing risk reduction with cultural and practical considerations. Regional variability in background PAH levels necessitates context-specific guidelines. A critical future direction is cumulative risk assessment that accounts for multiple exposure routes and co-exposures to other environmental contaminants [95,99,100].

The complexity of PAH behavior necessitates multidisciplinary approaches combining chemical principles, biological processes, and engineering solutions [101]. This review also highlights critical knowledge gaps and emerging research priorities, including PAH mixture toxicology, the behavior of nitro- and oxy-PAH derivatives, climate change impacts, sustainable remediation technologies, and refined risk assessment frameworks. Addressing these challenges requires continued interdisciplinary collaboration to protect human and ecological health from PAH contamination.

## 9. Conclusions

This review has integrated chemical, environmental and toxicological evidence on PAHs into a single entwined system.

Parent PAH carcinogenicity is mechanistically dominated by CYP450-mediated metabolic activation of HMW PAHs to bay-region diol-epoxide intermediates, which form stable and depurinating adducts with DNA. Combustion-derived PAHs grow predominantly by hydrogen-abstraction/acetylene-addition (HACA) kinetics at high temperatures, with hydrogen abstraction as the rate-limiting step and the OH radical as the most efficient abstractor. Physicochemical properties scale log-linearly with ring number and molecular mass, enabling robust prediction of environmental partitioning across compartments. Sorption to black carbon, biochar and combustion soot is the principal control on environmental bioavailability and persistence of HMW PAHs.

The current 16-priority-PAH regulatory framework underestimates total carcinogenic risk by an estimated factor of 2–5, owing to the neglect of alkylated homologs and oxy-/nitro-PAH derivatives that are often more potent than the parent compounds. Mixture toxicology remains poorly characterized; non-additive interactions in realistic environmental mixtures have not been systematically quantified. Dose–response relationships for non-cancer endpoints—cardiovascular, neurodevelopmental, immunotoxic—lack the quantitative basis needed for reference-dose derivation. The sequestration–bioavailability paradox (super-sorption to black carbon reduces uptake but extends persistence) is not yet resolved into operationally usable bioavailability metrics.

The relative contribution of HACA, HAVA, PAC, MAC and resonance-stabilized-radical chain reactions to PAH growth at intermediate temperatures (1000–1500 K) remains quantitatively uncertain. Heterogeneous diffusion limitations and viscous crust formation on combustion aerosols are not adequately parameterized in global transport models, leading to systematic underestimation of HMW-PAH atmospheric persistence. Temperature-dependent volatilization from legacy-contaminated soils and post-fire mobilization of PAH-rich ash under climate change scenarios are inadequately constrained by empirical data.

## Figures and Tables

**Table 1:** Selected U.S. EPA priority PAHs with structural characteristics and IARC carcinogenicity classifications. LMW = low molecular weight; HMW = high molecular weight. IARC Groups: 1 = carcinogenic to humans; 2A = probably carcinogenic; 2B = possibly carcinogenic; 3 = not classifiable.

Compound	Rings	MW (g/mol)	Classification	Carcinogenicity
Naphthalene	2	128	LMW	Possible (2B)
Acenaphthene	3	154	LMW	Not classified
Fluorene	3	166	LMW	Not classified
Phenanthrene	3	178	LMW	Not classified
Anthracene	3	178	LMW	Not classified
Fluoranthene	4	202	HMW	Possible (3)
Pyrene	4	202	HMW	Not classified
Benz[a]anthracene	4	228	HMW	Probable (2A)
Chrysene	4	228	HMW	Probable (2B)
Benzo[b]fluoranthene	5	252	HMW	Probable (2B)
Benzo[a]pyrene	5	252	HMW	Carcinogenic (1)
Dibenz[a,h]anthracene	5	278	HMW	Probable (2A)
Indeno[1,2,3-cd]pyrene	6	276	HMW	Probable (2B)

**Table 2:** Key physicochemical properties of selected PAHs at 25 °C. MW = molecular weight; VP = vapor pressure; MP = melting point. Data compiled from [5,7,8,9].

Compound	MW (g/mol)	Solubility (mg/L)	VP (Pa)	log K_OW_	MP (°C)
Naphthalene	128	31.0	10.4	3.37	80
Acenaphthene	154	3.8	0.29	3.92	95
Fluorene	166	1.9	0.09	4.18	116
Phenanthrene	178	1.1	0.02	4.57	101
Fluoranthene	202	0.26	1.2 × 10^−3^	5.22	111
Pyrene	202	0.13	6 × 10^−4^	5.18	156
Benz[a]anthracene	228	0.011	2.8 × 10^−5^	5.91	160
Chrysene	228	0.002	5.7 × 10^−7^	5.86	254
Benzo[a]pyrene	252	0.0016	7 × 10^−7^	6.04	179
Indeno[1,2,3-cd]pyrene	276	6.2 × 10^−5^	1.3 × 10^−8^	6.50	163

## Data Availability

All data included in the manuscript.
